# Longitudinal Replication Studies of GWAS Risk SNPs Influencing Body Mass Index over the Course of Childhood and Adulthood

**DOI:** 10.1371/journal.pone.0031470

**Published:** 2012-02-15

**Authors:** Hao Mei, Wei Chen, Fan Jiang, Jiang He, Sathanur Srinivasan, Erin N. Smith, Nicholas Schork, Sarah Murray, Gerald S. Berenson

**Affiliations:** 1 Department of Epidemiology, Tulane University, New Orleans, Louisiana, United States of America; 2 Tulane Center for Cardiovascular Health, Tulane University, New Orleans, Louisiana, United States of America; 3 Shanghai Children's Medical Center, Shanghai Jiao Tong University School of Medicine, Shanghai, China; 4 Department of Pediatrics and Rady's Children's Hospital, University of California at San Diego, School of Medicine, La Jolla, California, United States of America; 5 Scripps Genomic Medicine and Scripps Translational Science Institute, La Jolla, California, United States of America; Neocodex, Spain

## Abstract

Genome-wide association studies (GWAS) have identified multiple common variants associated with body mass index (BMI). In this study, we tested 23 genotyped GWAS-significant SNPs (p-value<5*10-8) for longitudinal associations with BMI during childhood (3–17 years) and adulthood (18–45 years) for 658 subjects. We also proposed a heuristic forward search for the best joint effect model to explain the longitudinal BMI variation. After using false discovery rate (FDR) to adjust for multiple tests, childhood and adulthood BMI were found to be significantly associated with six SNPs each (q-value<0.05), with one SNP associated with both BMI measurements: KCTD15 rs29941 (q-value<7.6*10-4). These 12 SNPs are located at or near genes either expressed in the brain (BDNF, KCTD15, TMEM18, MTCH2, and FTO) or implicated in cell apoptosis and proliferation (FAIM2, MAP2K5, and TFAP2B). The longitudinal effects of FAIM2 rs7138803 on childhood BMI and MAP2K5 rs2241423 on adulthood BMI decreased as age increased (q-value<0.05). The FTO candidate SNPs, rs6499640 at the 5 ′-end and rs1121980 and rs8050136 downstream, were associated with childhood and adulthood BMI, respectively, and the risk effects of rs6499640 and rs1121980 increased as birth weight decreased. The best joint effect model for childhood and adulthood BMI contained 14 and 15 SNPs each, with 11 in common, and the percentage of explained variance increased from 0.17% and 9.0*10^−6^% to 2.22% and 2.71%, respectively. In summary, this study evidenced the presence of long-term major effects of genes on obesity development, implicated in pathways related to neural development and cell metabolism, and different sets of genes associated with childhood and adulthood BMI, respectively. The gene effects can vary with age and be modified by prenatal development. The best joint effect model indicated that multiple variants with effects that are weak or absent alone can nevertheless jointly exert a large longitudinal effect on BMI.

## Introduction

Obesity, a major global public health concern, is a deleterious factor associated with different diseases, including cardiovascular events, type 2 diabetes mellitus, sleep -breathing abnormalities, and some cancers [1], [2]. The prevalence of obesity in children and adults has increased strikingly over the past decades, leading to an increased likelihood of serious obesity-related complications and decreased life expectancy [3]. Thus, reducing obesity prevalence in adults and preventing overweight from early childhood is of great importance in terms of public health.

The predisposition to obesity varies widely in the population and is partially genetically determined. Intrauterine growth, measured by birth weight, can influence the probability of suffering from obesity in future life [4], [5]. Heritability of the underlying genetic components ranges from 25% to 40% according to family studies, and from 50% to 80% according to twin studies. Longitudinal studies in twins have shown genetic effects on obesity through middle age (over the course of 43 years) [6]. Recent advances in genome-wide association studies (GWAS) have evidenced the association of common variants of several different genes with body mass index. Among these genes, FTO has been repeatedly identified [7]–[11], and KCTD15, TMEM18, MTCH2, and NEGR1 have been considered to confer obesity risk through effects in the central nervous system [9]. Novel functions of three other genes, FAIM2, MAP2K5, and TFAP2B, were also found to be associated with obesity development [11].

The longitudinal effects of these GWAS findings over the course of childhood and adulthood, however, have not been widely studied, and each variant can explain only a very small proportion of BMI variance. It is not yet clear if there are joint effects among these variants and if these effects are age-dependent and modifiable by birth weight. To address these questions, we conducted a longitudinal replication study in the Bogalusa Heart Study cohort, using BMI data collected from 1973 to the present. We previously identified the longitudinal effects of FTO tag variants associated with birth weight [12]. In this follow-up study, we will examine 23 additional GWAS-significant SNPs and explore their joint effects using a heuristic forward searching strategy.

## Materials and Methods

The Bogalusa Heart Study is a community-based study of the natural history of cardiovascular disease since childhood in the community of Bogalusa, Louisiana. The study is longitudinal, and the initial cross-sectional study began in 1973–1974. Subsequent cross-sectional surveys were conducted every 3–4 years during childhood and young adulthood. At present, seven major cross-sectional surveys of children aged 3–17 years and six of adults aged 18–44 years, who were previously examined as children, have been conducted. This enabled us to study the evolution of obesity over a 40-year lifespan from childhood through middle-age. Information on personal health and medication history was obtained from participants through questionnaires. Standard protocols approved by the Institutional Review Board of the Tulane University Health Sciences Center were used for the collection of all data [13], [14]. Written informed consent was obtained from the participants, or their parents (guardians) if the participants were children.

Participating subjects taken from the BHS for this replication study consist of 658 whites (308 males and 350 females). The birth weight data (kg) and gestational age (weeks) from menstrual history were retrieved from Louisiana State birth certificates. Their anthropometric measures were made in both childhood and adulthood. All examinations followed essentially the same protocols. Height and weight were measured twice to ±0.1 cm and to ±0.1 kg, respectively. The means of two independent measurements for height (in centimeters) and weight (in kilograms) were used to calculate BMI (kilograms per meter squared) as the measure of obesity. On average, study subjects were surveyed 4.3 times over childhood and 4.5 times over adulthood. Genotyping of BHS participants was based on the Illumina Human610 BeadChip and HumanCVD BeadChip (also referred to as IBC array), and completed in the laboratory of Genomic Medicine at the Scripps Research Institute in La Jolla, California [15]. Total 23 SNPs previously reported to be significantly associated with BMI in GWAS (p-value≤5*10^−8^) were selected for this replicated longitudinal study, based on PubMed literature review and NHGRI GWAS Catalog [16], [17].

Statistical analyses were performed using R (version 2.10.1). Linear modeling was used to examine the major effect of each polymorphism on birth weight, with gestational age and sex controlled as confounding covariates. Longitudinal effects of candidate SNPs on childhood (age<18 years) and adulthood (age≥18 years) BMI were examined by linear mixed model using the R package lme4, with birth weight and age as confounding covariates. A random effect of age was included to control for repeated measures of BMI at different ages. Interaction tests were conducted to measure the modifications of variant effects by age and birth weight. For all studies, both general and additive genetic models were applied, which respectively treat a SNP as a categorical factor and as a continuous variable coded as 0, 1, or 2 copies of a particular reference allele. The false discovery rate (FDR) was taken to adjust for multiple tests [18]–[20]. The FDR q-value of a variant association was defined as the smaller of two genetic models and a q-value≤0.05 was considered significant.

A heuristic forward search was proposed to identify the best joint effect model among candidate variants through the addition of SNPs one by one. At each forward step, the next SNP and its corresponding reference allele were identified by the criterion that the number of reference alleles over all added SNPs must have the minimum modified Akaike Information Criterion (AIC) [21] of the linear mixed model. The modified AIC is defined as: 2*(the number of SNPs+the number of non-SNP parameters) – 2*ln(*L*), where *L* is the maximized likelihood for the estimated model. The search will stop when the modified AIC of the best joint effect model cannot be further reduced in the next step. The BMI was permuted 10,000 times to generate distribution of a random best joint effect model and the empirical p-value was calculated as the percentage of minimized AIC≤observed minimized AIC.

## Results

Characteristics of participants at birth, first measurement during childhood, last measurement during adulthood, and p values for differences between genders are summarized in Table 1. Descriptions of 23 replicated SNPs are presented in Table 2. Hardy-Weinberg Equilibrium (HWE) was met for all SNPs in the study cohort. After adjustment for multiple tests, no significant associations were observed between candidate SNPs and birth weight (results not shown).

**Table 1:** Mean levels (standard deviation) of study variables by sex.

	Male (N = 308)	Female (N = 350)	Gender* Difference
Birth Registry Information			
Birth weight (kg)	3.47 (0.53)	3.33 (0.52)	P<0.001
Gestational Age (week)	39.77 (1.67)	39.81 (1.90)	P = 0.798
Childhood (first Measurement)			
Age (year)	9.92 (3.17)	9.66 (3.18)	P = 0.303
Weight (kg)	35.21 (15.21)	33.86 (14.26)	P = 0.532
Height (cm)	137.94 (19.74)	135.55 (19.11)	P = 0.070
BMI (kg/m^2^)	17.64 (3.41)	17.55 (3.45)	P = 0.890
Adulthood (Last Measurement)			
Age (year)	33 (4.67)	32.13 (4.90)	P = 0.206
Weight (kg)	89.34 (18.19)	72.31 (19.54)	P<0.001
Height (cm)	177.67 (6.39)	163.26 (6.23)	P<0.001
BMI (kg/m^2^)	28.26 (5.44)	27.11 (7.15)	P = 0.023

*Difference in birth weight was adjusted for gestational age; difference in weight, height and BMI was adjusted for age.

**Table 2:** Characteristic of candidate SNPs.

SNP	Genes	CHR	strand	Position	A1/A2	MAF	GWAS_P	Hetero-zygosity	HWE_P
rs2568958	NEGR1	1	+	72537704	G/A	0.38	1.00E-11	0.47	0.6
rs2815752	NEGR1	1	−	72585028	C/T	0.38	2.00E-22	0.47	0.6
rs1514175	TNNI3K	1	−	74764232	T/C	0.4	8.00E-14	0.48	1
rs2867125	TMEM18	2	−	612827	A/G	0.18	3.00E-49	0.3	0.35
rs7561317	TMEM18	2	+	634953	A/G	0.19	4.00E-17	0.3	0.13
rs13078807	CADM2	3	+	85966840	G/A	0.22	4.00E-11	0.35	1
rs7647305	ETV5	3	+	187316984	T/C	0.22	7.00E-11	0.35	0.55
rs13107325	SLC39A8	4	+	103407732	T/C	0.09	2.00E-13	0.16	1
rs987237	TFAP2B	6	+	50911009	G/A	0.17	3.00E-20	0.28	0.54
rs10968576	LRRN6C	9	+	28404339	G/A	0.3	3.00E-13	0.42	0.28
rs925946	BDNF	11	+	27623778	T/G	0.33	9.00E-10	0.44	0.48
rs6265	BDNF	11	−	27636492	A/G	0.19	5.00E-10	0.31	0.5
rs10767664	BDNF	11	+	27682562	T/A	0.2	5.00E-26	0.32	0.59
rs10838738	MTCH2	11	+	47619625	G/A	0.35	5.00E-09	0.46	0.65
rs7138803	FAIM2	12	+	48533735	A/G	0.37	2.00E-17	0.46	0.94
rs2241423	MAP2K5	15	+	65873892	A/G	0.22	1.00E-18	0.34	0.26
rs6499640	FTO	16	+	52327178	G/A	0.39	4.00E-13	0.48	0.19
rs1121980	FTO	16	−	52366748	T/C	0.42	4.00E-08	0.49	0.94
rs8050136	FTO	16	+	52373776	A/C	0.4	1.00E-47	0.48	1
rs571312	MC4R	18	−	55990749	T/G	0.23	6.00E-42	0.35	0.08
rs12970134	MC4R	18	+	56035730	A/G	0.27	1.00E-12	0.4	0.43
rs29941	KCTD15	19	−	39001372	T/C	0.31	7.00E-12	0.42	0.62
rs2287019	QPCTL	19	+	50894012	T/C	0.17	2.00E-16	0.29	0.81

Strand: + (sense strand) and − (antisense strand); Position (bp): It is based on coordinates of NCBI B36. A1/A2: Minor Allele/Major Allele; MAF: Minor Allele Frequency; GWAS_P: previously reported GWAS p-value; HWE_P: The p-value of Hardy-Weinberg Equilibrium test.

**Figure 1:**
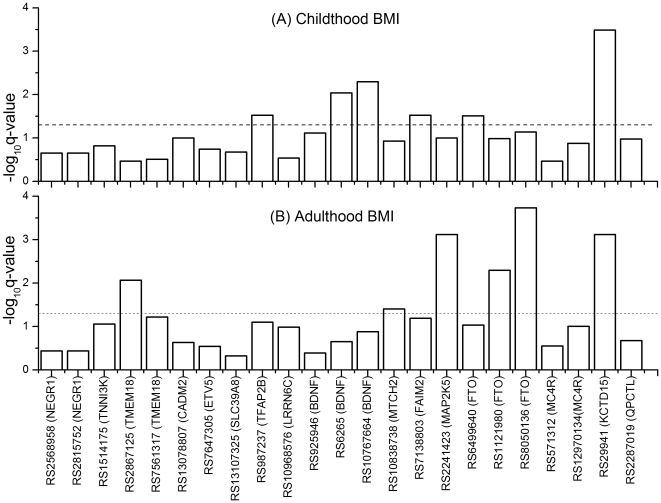
Association of candidate SNPs with repeated measures of body mass index (BMI), adjusted for sex, age, and birth weight. (**A**) **childhood BMI and** (**B**) **adulthood BMI.**

**Figure 2:**
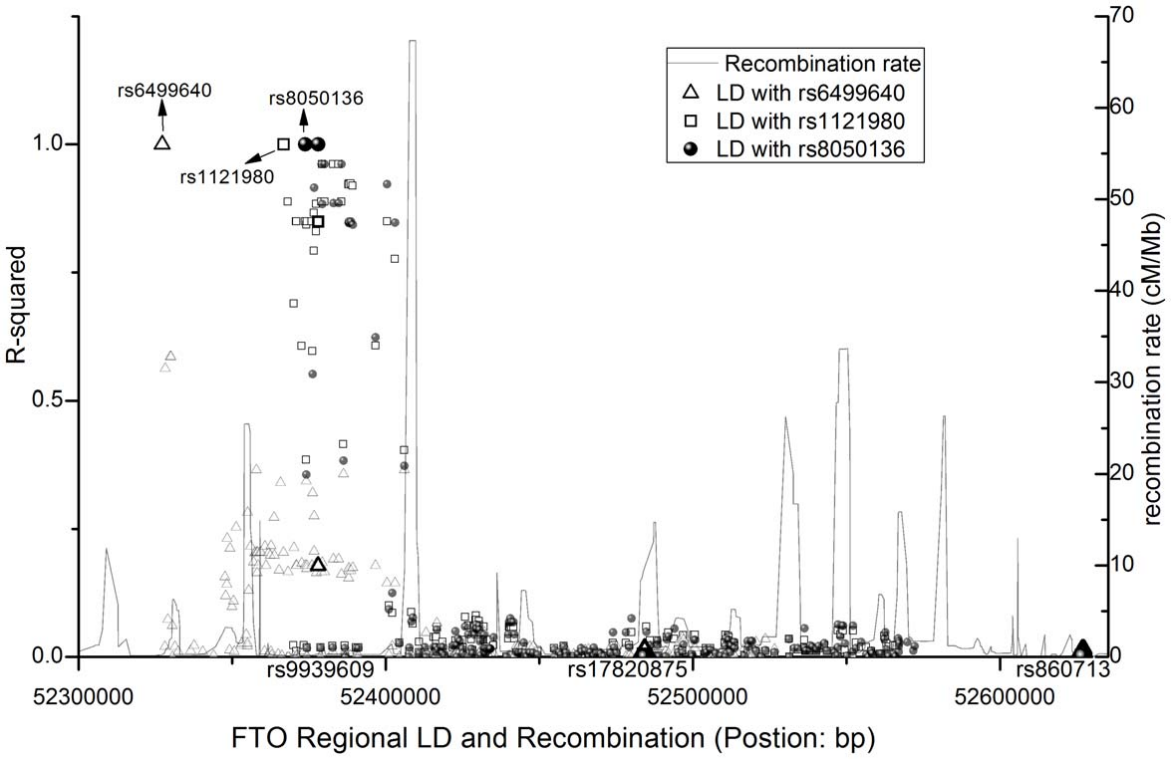
FTO regional linkage disequilibrium (LD) and recombination rate. The LD is from HapMap rel. 27 and the recombination rate is from HapMap rel. 22. SNP position is based on NCBI build 36 of the human genome.

Logarithm base 10 q-values are presented in Figure 1 for the association tests between candidate SNPs and longitudinal BMI adjusted for birth weight, age, and sex. Six SNPs, rs987237 (TFAP2B), rs6265 (BDNF), rs10767664 (BDNF), rs7138803 (FAIM2), rs6499640 (FTO), and rs29941 (KCTD15), were associated with longitudinal childhood BMI with q-values (p-values) of 0.03 (0.004), 0.009 (8.6*10^−4^), 0.005 (3.7*10^−4^), 0.03 (0.004), 0.03 (0.004), and 3.2*10^−4^ (6.6*10^−6^), respectively. Six other SNPs, rs2867125 (TMEM18), rs10838738 (MTCH2), rs2241423 (MAP2K5), rs1121980 (FTO), rs8050136 (FTO), and rs29941 (KCTD15), were associated with longitudinal adulthood BMI, with q-values (p-values) of 0.009 (7.5*10^−4^), 0.04 (0.007), 7.6*10^−4^ (2.8*10^−5^), 0.005 (3.8*10^−4^), 1.8*10^−4^ (2.5*10^−6^), and 7.6*10^−4^ (3.1*10^−5^), respectively. The FTO regional recombination rate (HapMap rel. 22) [22] and LD (HapMap rel. 27) for the three candidate FTO-gene SNPs found in this study are presented in Figure 2.

**Figure 3:**
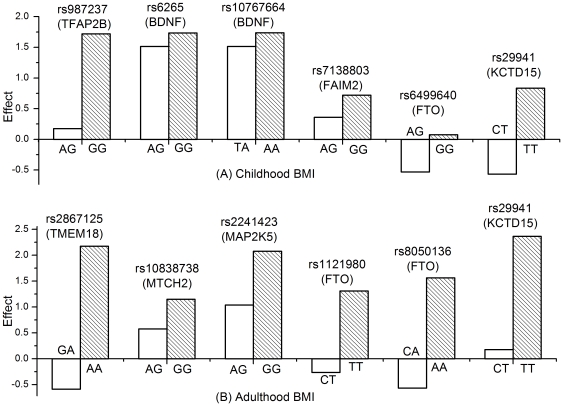
Major effects of genotypes for candidate SNPs on repeated measures of (A) childhood BMI and (B) adulthood BMI, adjusted for age and birth weight.

For each significant SNP, we compared the heterozygous genotype and the homozygous genotype with the higher BMI to the homozygous genotype with the lower BMI. The genotypes (effects) are as follows: for childhood BMI, AG (0.18) and GG (1.72) for rs987237, AG (1.51) and GG (1.74) for rs6265, TA (1.51) and AA (1.74) for rs10767664, AG (0.36) and GG (0.72) for rs7138803, AG (−0.53) and GG (0.07) for rs6499640, and CT (−0.57) and TT (0.83) for rs29941; for adulthood BMI, GA (−0.59) and AA (2.17) for rs2867125, AG (0.58) and GG (1.15) for rs10838738, AG (1.04) and GG (2.08) for rs2241423, CT (−0.26) and TT (1.31) for rs1121980, CA (−0.57) and AA (1.56) for rs8050136, and CT (0.18) and TT (2.37) for rs29941. The results are illustrated in Figure 3. General inheritance models were best for all SNPs except rs10838738, rs2241423, and rs7138803, for which additive inheritance models were best.

The significant interactions of SNP×age and SNP×birth weight are presented in Table 3. There were significant interactions between age and rs7138803 (FAIM2) for childhood BMI (q-value = 7.56*10^−4^, p-value = 2.46*10^−5^) and between age and rs2241423 (MAP2K5) for adulthood BMI (q-value = 0.02, p-value = 0.002) with adjustment for sex and birth weight. Each year of increase in age is estimated to decrease genotype AG and GG effects by 0.07 (β1) and 0.17 (β2) for rs7138803, respectively, and genotype AG and GG by 0.05 (β1) and 0.10 (β2) for rs2241423, respectively. There were also significant interactions between birth weight and rs6499640 (FTO) for childhood BMI (q-value = 0.037, p-value = 0.008) and between birth weight and rs1121980 (FTO) for adulthood BMI (q-value = 0.043, p-value = 0.009), with adjustment for age and sex. Each kilogram of increase in birth weight is estimated to decrease genotype AG and GG effects by 0.91 (β1) and 0.04 (β2) for rs6499640, respectively, and genotype CT and TT by 0.88 (β1) and 1.75 (β2) for rs1121980, respectively.

**Table 3:** Significant Birth Weight×SNP and Age×SNP interactions.

Interaction	Age Range (years)	ReferenceAllele	β1	β2	p-value	q-value
Age×rs7138803	<18	G	0.07	−0.17	2.46*10^−5^	7.56*10^−4^
Age×rs2241423	≥18	G	−0.051	−0.10	0.002	0.02
BW×rs6499640	<18	G	−0.91	−0.04	0.008	0.037
BW×rs1121980	≥18	T	−0.88	−1.75	0.009	0.043

BW: Birth Weight; β1: effect changes of heterozygous genotype for each one-year increase in age or one-kilogram increase in birth weight; β2: effect changes of homozygous genotype of reference allele for each one-year increase in age or one-kilogram increase in birth weight.

**Figure 4:**
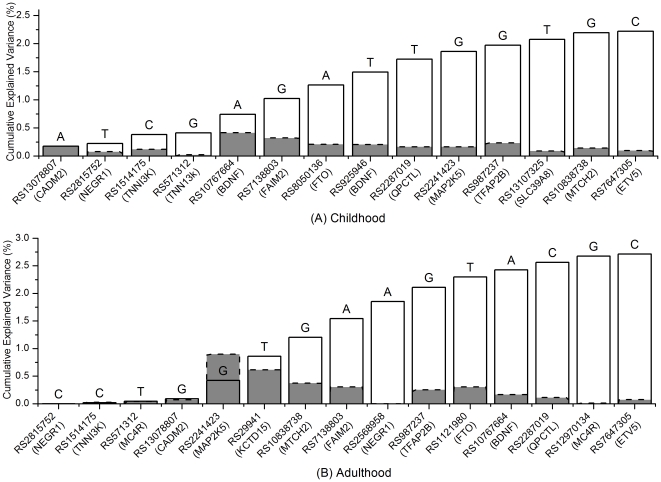
Percentage of cumulative BMI variance explained by sequentially added SNPs, shown from left to right, for (A) childhood BMI and (B) adulthood BMI. Solid line: percentage of cumulative explained BMI variance. Dashed line: percentage of single-variant explained BMI variance. The reference allele is indicated at the top of each column.

The best joint effect model for longitudinal BMI was generated by sequentially adding SNPs, with adjustment for age, sex, and birth weight; these results are presented in Figure 4. The best model for childhood BMI contained 14 SNPs and increasingly explained the variance, from 0.17% for the first added SNP (CADM2 rs13078807) to 2.2% for the last added SNP (ETV5 rs7647305). Every additional copy of the reference allele was estimated to increase BMI by 0.27, with an empirical p-value = 0. The interaction test showed that a one year increase in age decreased the risk variant effect on BMI by 0.01 (*p<0.05*). No significant interaction was observed between the best model and birth weight. The best model for adulthood BMI contained 15 SNPs and increasingly explained the variance from 9.0*10^−6^% for the first added SNP (NEGR1 rs2815752) to 2.7% for the last added SNP (ETV5 rs7647305). Each copy of the reference allele was estimated to increase BMI by 0.51, with an empirical p-value = 0. No significant interaction was observed between the best model and age or between the best model and birth weight (*p>0.05*).

## Discussion

Recent genome-wide association studies have identified several variants associated with BMI. We previously confirmed that FTO tag SNPs, including rs9939609, were associated with longitudinal adulthood BMI [12]. In this study, we extended the test to 23 additional GWAS-significant SNPs that were genotyped in the Bogalusa Heart Study cohort. BMI was repeatedly measured from age 3.32 to18 years for childhood, and from 18 to 45.30 years for adulthood. In contrast to other studies that focus on one measure of BMI per subject or measures at fixed ages, the repeated measures of BMI at broadly varied ages should cover more useful variation in the development of obesity determined by genetic components. Moreover, although the study sample included only 658 white subjects, multiple measures of BMI at different ages per subject increased the sample size of BMI observations to 2,708 for childhood and 2,844 for adulthood. By applying QUANTO [23] software, we confirmed that this study has 80% power to detect genetic variant or interaction effects, explaining 2.3% of the variance of the trait, given the following conditions: sample size of 658, type I error of 0.002 based on Bonferroni adjustment for 23 variants, and an additive genetic model. However, the study can detect much smaller variant or interaction effects than estimated here, because the longitudinal repeated measures serve to increase the number of observations about 4-fold, and both additive and general genetic models were examined, in order to reduce the risk of missing risk variants.

In this study, none of the 23 candidate variants was associated with birth weight (results now shown). The study observed that variants near or at certain genes were associated with BMI at different age ranges. Variants near or at BDNF (rs6265 and rs10767664), FAIM2 (rs7138803), TFAP2B (rs987237), and FTO (rs6499640) were associated with childhood BMI. Variants near or at MAP2K5 (rs2241423), MTCH2 (rs10838738), TMEM18 (rs2867125), and FTO (rs1121980 and rs8050136) were associated with adulthood BMI. Meanwhile, variants near or at KCTD15 rs29941 were associated with both childhood and adulthood BMI. Among these genes, BDNF [7], [24], [25], KCTD15 [9], TMEM18 [7], [26], and FTO [7], [27] were highly expressed in the central nervous system, and FAIM2 [28], TFAP2B [29], MAP2K5 [30], and MTCH2 [9], [31] were involved in cell apoptosis and proliferation. These findings suggested that genes implicated in pathways related to neural development and cell metabolism can have long-term effects on obesity development, and that the genes underlying childhood and adulthood BMI can be different.

FTO, a gene on chromosome 16, is highly expressed in the brain, and may influence feeding regulation [7]. The associations of FTO variants with BMI have been widely replicated in different studies. In this study, we confirmed that the FTO effects were longitudinal, influencing both childhood and adulthood BMI, repeatedly measured from 3–48 years of age. The results also indicated that the effects of FTO on obesity, due to a reduction in sensitivity of the appetite control system [32], can start from a young age, and can even be modified during prenatal development. However, no previous study provided the kind of direct evidence for an association between FTO and birth weight that was observed in this study. The FTO candidate SNPs (rs6499640, rs1121980, and rs8050136) were located at the 5′ end, upstream of the tag SNPs (rs9939609, rs17820875, and rs860713) that were explored previously [12] (Figure 2). These findings indicate that different regions of FTO are involved in childhood and adulthood obesity development; the upstream variant (rs6499640) was found to influence childhood BMI, and the downstream variants (rs1121980, rs8050136, rs9939609 [12], rs17820875 [12], and rs860713 [12]) were found to influence adulthood BMI. The risk variants for adulthood BMI, rs1121980, rs8050136, and rs9939609 [12], located between the first two exons of FTO, had strong pairwise LD *(r^2^≥0.85*). Regional recombination and LD plots show that they are located at the LD block between the upstream (25.46 cM/Mb) and downstream recombination hotspots (67.32 cM/MB). In contrast, the other three SNPs were located at or around the hotspot regions.

The interaction study showed that the longitudinal effects of FAIM2 rs7138803 on childhood BMI and MAP2K5 rs2241423 on adulthood BMI depend on age. The risk effects of the rs7138803 allele *G* (q-value = 7.6*10^−4^, p-value = 2.5*10^−5^) and the rs2241423 allele *G* (q-value = 0.02, p-value = 0.004) were significantly reduced with increasing age. Both FAIM2 [28] and MAP2K5 [30] regulate cell metabolism. The results indicate that age-dependent genetic pathways related to cell apoptosis and proliferation can regulate longitudinal BMI changes. This interaction study also showed that effects of FTO rs6499640 on childhood BMI and rs1121980 on adulthood BMI were associated with birth weight. There was a significant negative association between birth weight and risk genotype effect, and our findings indicate that low birth weight increases the risk effect of rs6499640 *GG* and rs1121980 *TT*. Birth weight is an indicator of intrauterine development of the fetus. There is evidence that intrauterine development can influence the risk of later onset obesity [3]. The findings of the present study suggest that fetal nutrition can change the predisposition to obesity by modifying these obesity-related gene effects.

SNPs in high LD and the tests of same variants with different genetic models can all cause correlated results. To efficiently control for correlated multiple tests, we selected the FDR method to estimate the fraction of false positives among all tests (q-value). In contrast to the false positive rate (p-value) a measure of the rate of null associations deemed significant, the FDR cutoff point of q = 0.05 ensures that the error rate for findings deemed significant, but that are actually false positives, is smaller than 5%. In this study, both general and additive genetic models were used to examine the longitudinal effects of candidate SNPs. This strategy will improve the potential to identify inherited risk variants. For example, tests of FTO variants (rs6499640, rs1121980, and rs8050136) based on the general genetic model presented much stronger associations with longitudinal BMI (q-value = 1.8*10^−4^∼0.03) than tests based on the additive genetic model (q-value = *0.07∼0.10*). Estimates of genotype effects based on the general model indicated that the pattern of inheritance is closer to codominance (Figure 3).

Joint effects of multiple common variants at GWAS are often estimated as a cumulatively additive effect for the number of risk alleles, using weighted or unweighted methods [27], [33], [34]. However, these methods generally require that all variants are independently associated with risk [33]. In addition, because of allele heterogeneity, the risk alleles may not be the same in different populations. We therefore proposed the forward heuristic search method to identify the best joint effect model and the corresponding risk variants among the candidate variants. This method does not require mutual independence of the candidate variants. It attempts to identify a list of variants that are sequentially selected to make the best joint effect model and to continuously improve prediction for target phenotype based on minimization of AIC. In contrast to searching for SNPs that have the largest effects at each step, the forward searching step minimizing AIC has the advantage of maximally explaining BMI variation by analyzing joint effects of multiple common variants and confounding effects of non-genetic covariates together. For example, in the best joint effect model search analysis of longitudinal childhood BMI, rs2815752, the first selected SNP, explained a much smaller percentage of BMI variance (9.0*10^−6^%) than rs2241423, which explained the largest percentage of BMI variance (0.90%). However, together with the three covariates, age, sex, and birth weight, rs2815752 best predicted the longitudinal BMI with the minimized AIC. The p-value for the best joint effect model is based on a permutation test that also controls for potential correlation among candidate variants.

Population structure and heterogeneity are critical issues in causing false-positive associations. In this study, all subjects were randomly selected from the white population living in the rural community of Bogalusa, Louisiana. All 23 variants have frequencies close to their frequencies in the HapMap white population (CEU) and meet Hardy-Weinberg equilibrium. All of the subjects had lived in a small town and in the same neighborhood since childhood, and environmental factors within the community were potentially homogeneous [35], [36]. Thus, confounding effects due to unknown environment factors, genetic heterogeneity due to different ethnicity, and false-positive associations due to biased selection were reduced in this study. However, due to the restriction of available data, we did not consider socioeconomic status, physical activity, calorie intake, smoking, diabetes, hypertension, or family history for their potential influences on this longitudinal genetic study. Residual confounding due to these factors and the potential existence of unknown subpopulation stratification within the studied white population may affect our findings. The influence of these factors on the findings may require evaluation in follow-up studies.

In summary, we performed a longitudinal study to examine the long-term effects of candidate SNPs that had been previously reported as BMI-related risk variants in published GWAS. The present study confirmed that risk variants of genes implicated in pathways related to neural development and cell metabolism exert major longitudinal effects on BMI. We also found that there are different sets of risk variants associated with childhood and adulthood BMI; that the effects of FAIM2 and MAP2K5, both genes from a cell metabolism-related pathway, vary significantly with age; that FTO influences both childhood and adulthood BMI; that effects on obesity through appetite changes can be modified during prenatal development; and that multiple variants with weak or absent effects alone can jointly exert a large longitudinal effect. These findings provide new insights into biological processes influencing obesity development over the course of childhood and adulthood, and will help to guide future study of genetic pathways related to obesity development.
